# Educational impact of a structured simulation-based orthopedic training program on technical and non-technical competency development: a prospective pre–post study

**DOI:** 10.25122/jml-2026-0021

**Published:** 2026-02

**Authors:** Ionut Dudau, Dumitru Sutoi, Bogdan Chiu, Raluca Radbea, George Marin, Anda Nicoleta Ciontos, Vlad Mulcutan-Chis, Maria Sutoi, Ovidiu Alexandru Mederle, Bogdan Nicolae Deleanu

**Affiliations:** 1 Doctoral School, Faculty of General Medicine, Victor Babes University of Medicine and Pharmacy Timisoara, Timisoara, Romania; 2 Department of Surgery, Emergency Discipline, Victor Babes University of Medicine and Pharmacy, Timisoara, Romania; 3 Emergency Municipal Clinical Hospital, Timisoara, Romania; 4 Victor Babes University of Medicine and Pharmacy, Timisoara, Romania; 5 Department of Cardiology, Institute of Cardiovascular Diseases, Timisoara, Romania; 6 Pius Brinzeu Emergency County Hospital, Timisoara, Romania

**Keywords:** simulation-based learning, surgical skills training, technical skills acquisition, pre-post educational intervention, medical students, non-technical skills

## Abstract

Contemporary orthopedic education faces reduced clinical exposure, increasing procedural complexity, and growing emphasis on patient safety. Simulation-based learning (SBL) offers a structured alternative to traditional apprenticeship models, enabling deliberate practice and competency-based assessment. This study evaluated the educational impact of a structured, hands-on, simulation-based orthopedic workshop on the development of technical and non-technical competencies among medical students. We conducted a prospective pre–post interventional study including 70 medical students across all 6 years of training. Participants completed pre- and post-intervention assessments evaluating self-confidence in musculoskeletal trauma management, perceived technical skills, non-technical competencies (communication, teamwork, situational awareness), motivation toward orthopedics, and theoretical knowledge through a 10-item multiple-choice questionnaire. Statistical analysis employed non-parametric testing (Wilcoxon signed-rank, Mann–Whitney U, Kruskal–Wallis). Effect sizes were calculated using r = Z/√N, with values > 0.5 indicating large effects. Internal reliability was assessed using Cronbach’s alpha. Significant improvements were observed across all evaluated domains (all *P* < 0.001), with large effect sizes (r > 0.5). Confidence in musculoskeletal trauma management demonstrated the greatest increase (median 4 pre-workshop vs. 9 post-workshop). Theoretical knowledge improved in most domains. Internal consistency of the assessment instrument was excellent (Cronbach’s alpha 0.896–0.961). Senior students demonstrated higher baseline and post-intervention scores compared to junior cohorts (*P* < 0.05). No significant gender-based differences were identified, except in procedural risk recognition (*P* < 0.001). Participation in a structured simulation-based orthopedic workshop was associated with meaningful multidimensional educational gains, including enhanced perceived technical competence, strengthened non-technical skills, increased confidence, and improved knowledge acquisition.

## Introduction

Patient safety, the primary concern of the clinician, together with the increasing constraints on clinical training time and the growing complexity of modern surgical procedures, characterizes the current transformation in orthopedic surgical education. Traditional apprenticeship-based models, such as “see one, do one, teach one” [1,2], are increasingly being challenged, particularly in developing technical competence while minimizing patient risk and ensuring standardized skill acquisition [3,4]. Optimizing educational strategies through simulation-based learning (SBL) offers a structured, reproducible alternative that supports the acquisition and refinement of surgical skills in a controlled environment. In orthopedic surgery, simulation modalities include task trainers, cadaveric laboratories, virtual reality simulators, and augmented reality platforms [5,6]. These approaches enable objective assessment of technical performance, facilitate deliberate practice, and allow repeated exposure to complex procedures without patient risk, thereby contributing substantially to competency development [7].

A growing body of evidence supports the effectiveness of simulation-based training in enhancing technical performance in orthopedic surgery. Improvements in execution quality, procedural accuracy, economy of movement, and the reduction of technical errors have been demonstrated following structured simulation curricula [8,9]. Randomized controlled trials in arthroscopy report significantly superior performance outcomes among residents trained using simulation compared with those trained exclusively through traditional models [10]. Similarly, in spine surgery, simulation-based training for pedicle screw placement has been associated with increased placement accuracy and improved instrument handling, underlining its value in technically demanding procedures [11].

Beyond improvements in isolated technical skills, simulation-based curricula have shown measurable benefits in procedural flow, time efficiency, and error reduction across orthopedic subspecialties. Meta-analytic evidence confirms that simulation enhances objective performance metrics, including task completion time and global rating scale scores, when compared to conventional training strategies [12]. Structured programs incorporating deliberate practice principles further demonstrate sustained skill acquisition and improved operative readiness [13,14]. In arthroscopy training specifically, randomized trials describe enhanced triangulation skills, improved camera navigation, and fewer iatrogenic errors among trainees exposed to simulator-based practice [15,16].

In spine and trauma surgery, simulation has demonstrated particular relevance in high-risk procedures such as pedicle screw placement and fracture fixation techniques. Studies evaluating virtual reality and navigation-assisted simulation models report higher technical accuracy, improved spatial orientation, and better instrument coordination among trained participants [17,18]. Broader educational research in simulation science reinforces that repetitive, feedback-driven practice not only accelerates skill acquisition but also enhances long-term retention and supports transfer of competencies into clinical settings [13,19].

Despite these promising findings, a critical appraisal of SBL in orthopedic education remains necessary to define its precise educational contribution. While systematic reviews and meta-analyses confirm improvements in performance metrics, heterogeneity in study design, variability in outcome measures, and limited long-term follow-up constrain definitive conclusions regarding sustained clinical transfer and patient-level outcomes [19,20]. Furthermore, important questions remain regarding cost-effectiveness, curricular standardization, optimal integration into residency programs, and the level of fidelity required to achieve meaningful educational gains [21]. Emerging evidence suggests that instructional design, structured feedback, and deliberate practice may exert greater influence on learning outcomes than technological sophistication alone [22]. Consequently, simulation should be embedded within competency-based curricula aligned with validated assessment frameworks rather than implemented as an isolated educational tool [23]. Collectively, while current evidence supports the educational value of SBL, its long-term contribution to surgical excellence and patient safety depends on systematic curricular integration, standardized assessment methodologies, and high-quality longitudinal research.

This study aimed to determine whether a structured, hands-on, simulation-based orthopedic workshop produces measurable improvements in technical performance perception, non-technical competencies, self-confidence in trauma management, and theoretical knowledge among medical students, as assessed through a validated pre–post educational evaluation framework. Additionally, the study sought to quantify effect sizes associated with competency gains, evaluate internal reliability of the assessment instrument, identify variability in outcomes across different stages of medical training, and explore demographic influences on perceived competence development. We hypothesized that a structured simulation-based educational intervention integrating fracture management and basic orthopedic surgical techniques would result in large effect-size improvements in both technical and non-technical competency domains, independent of gender and proportional to prior training level.

## Material and Methods

### Study design

This study employed a pre–post interventional educational design to evaluate the impact of a hands-on orthopedic workshop on participants’ self-confidence, non-technical skills, perceived practical competence, knowledge acquisition, and motivation.

An original questionnaire was developed to align directly with the specific objectives and multidimensional structure of the workshop. The questionnaire was created under the careful supervision of medical education experts to meet the criteria that we wanted to evaluate in the study. Given that no such questionnaire is available in the existing literature, we created a new one to meet the aim of this study. Existing validated instruments are primarily designed for residency-level surgical assessment and do not comprehensively capture early-stage competency development across technical, cognitive, and non-technical domains in undergraduate learners. Cronbach’s alpha was used to assess the questionnaire’s internal consistency. The instrument demonstrated excellent internal consistency, supporting its reliability in this study.

### Participants

The study included medical students from different training years who voluntarily participated in a practical orthopedic workshop. Participation in both the workshop and the evaluation process was optional. All participants were informed about the educational and research objectives of the study before enrolment.

### Workshop design and educational intervention

The educational intervention consisted of a structured, hands-on orthopedic workshop, designed to integrate theoretical concepts with practical skill acquisition in a simulated learning environment.

The workshop was divided into two main components:

#### Part I – Fracture first aid and immobilization

The first component focused on initial fracture management and first aid principles. Participants were introduced to fundamental concepts of fracture assessment and first aid, indications and principles of fracture immobilization, the structure, indications, and application principles of plaster splints and plaster casts. A live demonstration of plaster splint application was provided by the instructors, followed by supervised hands-on practice, during which participants individually performed plaster splint application.

#### Part II – Basic orthopedic surgical techniques

The second component addressed introductory orthopedic surgical techniques for both traumatic and degenerative musculoskeletal conditions. This session included presentation of basic orthopedic surgical instruments, simulation of surgical skin incision, dissection of anatomical layers following correct surgical planes, elevation of a simulated fracture site, simulation of fracture fixation using plates and screws, wound closure techniques, including simple interrupted sutures, running sutures, continuous sutures, surgical clips, and application of appropriate wound dressings.

The workshop emphasized step-by-step procedural learning, adherence to safety principles, and the development of both technical and non-technical skills, including communication, situational awareness, and task management.

### Educational materials

Educational materials included plaster splint materials, basic orthopedic surgical instruments, synthetic models and simulation materials for fracture fixation and suturing, and standardized wound-dressing supplies.

### Outcome measures

Participants completed a pre-test (administered before the workshop) and a post-test (administered immediately after the workshop).

### Self-assessment questionnaire

A structured questionnaire using Likert-type scale items (1–5) was used to assess: self-confidence in orthopedic trauma management, non-technical skills (communication, teamwork, situational awareness), perceived practical competence, motivation, and interest in orthopedics and trauma care.

### Knowledge assessment

A 10-item multiple-choice questionnaire (MCQ) was administered before and after the workshop to evaluate theoretical knowledge related to fracture first aid and immobilization, basic orthopedic surgical principles, osteosynthesis, and suturing techniques. The 10 MCQs are available in Supplementary File S1.

Supplementary File S1

### Data analysis

Data were managed using Microsoft Excel 2024 and analyzed using JASP version 0.95.4. Quantitative variables are presented as medians and interquartile ranges (IQR). The normality of data distribution was assessed using the Shapiro-Wilk test; as the data deviated significantly from a normal distribution (*P* < 0.05), non-parametric tests were employed.

Pre- and post-intervention scores were compared using the Wilcoxon signed-rank test. Effect sizes were calculated using the formula $Z /√N, where r > 0.5 was considered a large effect. For independent group comparisons, the Mann–Whitney U test was used for two groups, and the Kruskal–Wallis test was applied for comparisons involving more than two groups. Internal consistency of the survey instrument was evaluated using Cronbach’s alpha both before and after the workshop. Statistical significance was defined as *P* < 0.05.

## Results

The final database contained responses from 70 students. The number of men and women participating in the workshop is almost identical: 36 (51.43%) men and 34 (48.57%) women (Table 1). All six study years in the Romanian medical university system are represented, with 4^th^-year students being the most (31.43%). The median age was 22.5, with an interquartile range (IQR) of 19.5–22.5. To assess test consistency, Cronbach’s alpha was calculated for the questions on confidence, technical competencies, and non-technical skills, both for the test administered before and after the workshop. For the pre-workshop internal consistency, the values for the alpha were 0.961, 0.950, and 0.942. For the post-intervention evaluation, the values obtained were 0.919, 0.928, and 0.896, proving excellent internal consistency in the questions administered before and after the workshop.

**Table 1:** Demographic characteristics

Variable	*n* (%) or median (IQR)
**Age ^b^**	22.5 (19.5-25.5)
**Year of study**	
Year I	13 (18.57%)
Year II	9 (12.86%)
Year III	10 (14.29%)
Year IV	22 (31.43%)
Year V	3 (4.29%)
Year VI	13 (18.57%)
**Gender**	
Men	36 (51.43%)
Women	34 (48.57%)

b For age, the median (IQR) was displayed, with data non-parametric

(*P* = 0.011)

*n* = 70 total participants

Table 2 presents Likert-type responses before and after the workshop, reported as median (IQR). Pre-post differences were assessed using the Wilcoxon signed-rank test. Statistically significant improvements were observed across all evaluated domains (all *P* < 0.001), with large effect sizes (r > 0.5).

**Table 2:** Descriptive statistics

Category		Median (IQR) pre WKS	Median (IQR) post WKS	Statistic (W)	*P* value (*n* = 70)	Effect size ^®^
**CONFIDENCE**	Confidence in providing first aid for a suspected fracture	5 (3–7)	9 (7–10)	5	<0.001*	0.81
	Confidence in assessing traumatic limb injury	5 (3–7)	9 (8–10)	4.5	<0.001*	0.81
	Confidence in selecting the fracture immobilization method	5 (2–8)	9 (7–10)	10.5	<0.001*	0.80
	Confidence in applying plaster splint under supervision	5 (3–7)	9 (7–10)	35	<0.001*	0.78
	Confidence in managing musculoskeletal trauma	4 (1.5–6.5)	9 (8–10)	17	<0.001*	0.80
**TECHNICAL COMPETENCES**	Knowledge of plaster splint application steps	5 (2–8)	9 (7–10)	2.5	<0.001*	0.78
	Ability to identify immobilization mistakes	5 (2–8)	9 (7–10)	17	<0.001*	0.81
	Understanding of surgical wound management principles	5 (1–9)	9 (7–10)	23	<0.001*	0.79
	Understanding of basic surgical incision and dissection	5 (2.25–7.75)	9 (7.25–10)	9	<0.001*	0.81
	Understanding fracture reduction and stabilization	5 (2–8)	9 (7–10)	14	<0.001*	0.84
	Understanding osteosynthesis with plates and screws	5 (3–7)	9 (7.25–10)	20.5	<0.001*	0.82
**NON-TECHNICAL SKILLS**	Ability to assist in orthopaedic procedures under supervision	5 (3–7)	9 (7–10)	13.5	<0.001*	0.83
	Situational awareness during procedures	5 (3–7)	9 (7–10)	17.5	<0.001*	0.83
	Recognition of procedural risks	5 (1–9)	9 (7–10)	0	<0.001*	0.82
	Confidence in effective team communication	5 (1–9)	9 (8–10)	10	<0.001*	0.80
	Confidence in asking for help	6 (2–10)	9 (8–10)	41.5	<0.001*	0.75
	Workshops are essential for medical education.	2 (2–10)	10 (9–10)	16.5	<0.001*	0.71

* Statistically significant, *P* < 0.05, Wilcoxon Signed rank test

The greatest change was observed in confidence in managing musculoskeletal trauma, with the median increasing from 4 (IQR 1.5–6.5) pre-workshop to 9 (IQR 9–10) post-workshop. A substantial increase was also observed for the perceived necessity of practical workshops in medical education, with median scores rising from 7 (IQR 2–10) to 10 (IQR 9–10).

Overall, all domains assessed demonstrated significant improvements following the intervention.

**Figure 1:**
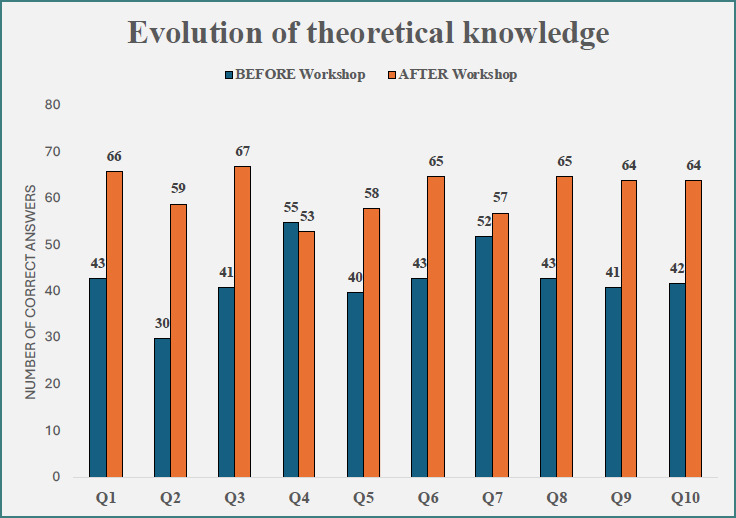
Evolution of theoretical knowledge

Theoretical knowledge was assessed using identical tests administered before and after the workshop. The number of correct responses increased for most theoretical domains following the intervention. The only domain that did not demonstrate improvement was anatomical considerations relevant to surgery (Q4), where 55 students answered correctly before the workshop, compared with 53 after the workshop. The overall change in theoretical performance is presented in Figure 1. All questions can be found in the Supplementary File S1.

Differences in Likert-scale responses across study years were analyzed using the Kruskal–Wallis test. Statistically significant differences between study years were identified for 17 variables related to confidence, risk recognition, and trauma management (*P* < 0.05). The biggest differences were observed for musculoskeletal trauma management confidence (H = 21.55, *P* < 0.001), appropriate method selection (H = 14.57, *P* = 0.12), and procedural risk recognition (H = 14.49, *P* = 0.013).

**Figure 2:**
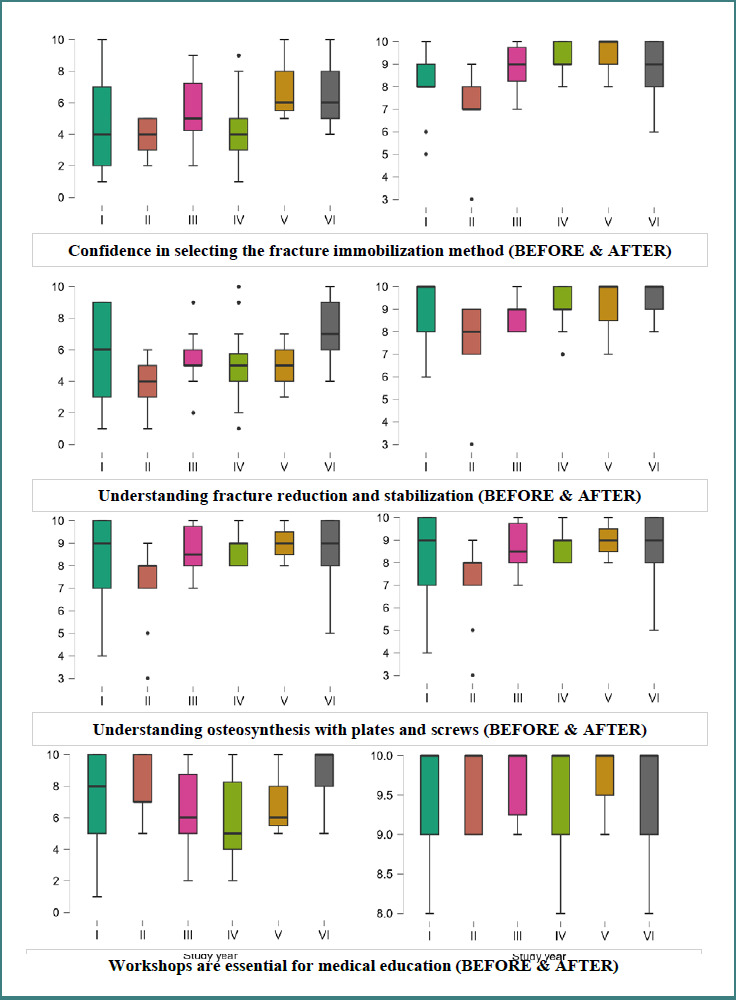
Comparative analysis between study years

Post-hoc pairwise comparisons using the Bonferroni correction revealed that senior students demonstrated significantly higher scores than in earlier study years across multiple domains. The most consistent differences were observed between Year II and Year VI students, particularly in musculoskeletal trauma management confidence, understanding of fracture reduction, and appropriate selection of treatment methods (adjusted *P* < 0.05). A before-and-after comparison for a few of the parameters analyzed is presented in Figure 2.

In the post-workshop evaluation, participants reported high levels of satisfaction across all evaluative domains. Post-workshop analysis revealed a median score of 10 across the following categories: desire for future workshop participation, utility of instructor feedback, and efficacy of the hands-on learning approach. Objectives were clearly defined, receiving a median score of 9 (IQR: 8–10).

The perceived importance of medical workshops increased significantly following the intervention. The proportion of participants assigning a maximum score increased from 34.28% at baseline to 61.42% post-workshop. A comparative analysis using the Mann–Whitney U test revealed no significant gender differences across most variables. However, a significant divergence was observed in procedural risk recognition, with male participants reporting higher confidence (U = 880.5, *P* < 0.001).

## Discussion

The present study demonstrates that a structured, simulation-based orthopedic workshop produced statistically significant and educationally meaningful improvements across all evaluated domains. The magnitude of change, reflected by large effect sizes (r > 0.5), suggests substantial educational benefit. Similar large effect sizes have been reported in meta-analyses evaluating technology-enhanced simulation in health professions education, confirming that structured simulation interventions generate meaningful improvements in knowledge and skill acquisition [24,25]. The most pronounced improvement was observed in confidence related to musculoskeletal trauma management. Confidence gains following simulation exposure have been consistently reported across surgical and procedural disciplines, particularly when learners are allowed to engage in deliberate practice within psychologically safe environments [26,27]. This supports the rationale presented in the introduction, which holds that simulation may compensate for reduced clinical exposure and for increasing procedural complexity.

Our findings are consistent with systematic reviews demonstrating that simulation-based education improves both technical and behavioral performance metrics [28,29]. In orthopedic surgery specifically, arthroscopy simulation studies have shown significant improvements in dexterity, triangulation, and procedural efficiency compared with traditional observational training models [30,31]. Moreover, transfer of simulator-acquired skills to the operating room has been demonstrated in controlled studies, strengthening the argument for simulation integration into structured curricula [32]. Importantly, the observed gains in non-technical domains—including communication, situational awareness, and task prioritization—align with literature emphasizing the critical role of non-technical skills in surgical safety and performance [33,34]. Modern surgical education increasingly recognizes that technical proficiency alone is insufficient; effective teamwork and cognitive load management are essential components of operative success.

The high Cronbach’s alpha values observed in our study indicate excellent internal consistency of the assessment instrument. This methodological robustness strengthens the validity of the findings. Educational research underscores that reliable assessment tools are fundamental for competency-based medical education frameworks and longitudinal skill tracking [35]. The progressive differences observed across study years are also consistent with staged competency development models, which describe increasing clinical reasoning sophistication and risk recognition across training levels [36]. Notably, simulation has been shown to accelerate early-stage learning curves, particularly when structured feedback and deliberate practice principles are incorporated [37].

The improvement in theoretical knowledge following the workshop reinforces the concept that simulation-based learning enhances cognitive integration rather than functioning solely as a psychomotor exercise. Studies evaluating blended simulation curricula demonstrate that combined theoretical instruction and hands-on practice improve retention and conceptual understanding more effectively than didactic instruction alone [38,39]. The lack of improvement in one anatomical domain may represent a ceiling effect or insufficient cognitive reinforcement, a phenomenon previously described in simulation literature when procedural focus outweighs anatomical contextualization [40].

Simulation-based curricula have been associated not only with immediate skill acquisition but also with improved long-term retention and reduced procedural error rates [41,42]. Furthermore, evidence suggests that structured simulation improves patient-level outcomes in selected procedural domains, although high-quality longitudinal studies remain limited [43]. The findings of this study support early integration of simulation within undergraduate curricula. Early exposure to structured orthopedic skill development has been associated with increased specialty interest and improved preparedness for surgical clerkships [44,45]. The Internet of Surgical Things (IoST) represents a significant advancement in telesurgery that can be used to teach students remotely. For example, principles of Image-Guided Surgery (IGS) use tracking devices, alongside pre- or even intra-operative imaging, to aid the surgeon in the hospital [46]. These methods can be used to support remote orthopedic training, so that students have direct access to better imaging of the surgery, a better understanding of the anatomy, and the surgical steps taken, all essential aspects of surgical understanding. Another interesting way to teach anatomy and surgical skills is to use 3D models to aid learning. For example, in cardiac medicine, the turning of digital models into physical objects has been used as an educational tool for students and healthcare professionals [47]. In orthopedics, the same concepts can be applied to teach the anatomy of specific body parts, congenital defects, or other complex anatomical challenges a student might encounter in their learning.

### Limitations and future directions

Several limitations should be acknowledged. The single-arm pre–post design without a control group limits causal inference, as improvements cannot be attributed exclusively to the intervention. In addition, reliance on self-reported measures may introduce perception bias, and perceived competence does not necessarily correspond to objectively validated procedural performance. Outcomes were assessed immediately after the workshop, precluding evaluation of long-term retention and transfer to clinical practice—an issue frequently highlighted in simulation research [48]. Another limitation of this study is the limited sample size and the low representation of certain study years. This limitation can introduce bias into the statistical analysis or increase the entropy of the results. Furthermore, the study did not assess cost-effectiveness or resource implications, which are important considerations for large-scale curricular implementation of simulation-based programs [49]. Future studies should incorporate randomized controlled designs, objective performance metrics, longitudinal follow-up, and economic evaluation frameworks to strengthen evidence for sustained clinical impact. These findings provide evidence to support the formal integration of simulation-based learning into undergraduate medical curricula, enabling universities to better introduce this approach into teaching. Further studies can be conducted to validate this teaching instrument and track students’ longitudinal progression across multiple years of training.

## Conclusion

This study demonstrates that a structured, simulation-based orthopedic workshop can produce significant multidimensional educational benefits among medical students. Participation in the intervention was associated with substantial improvements in self-confidence related to musculoskeletal trauma management, perceived technical competence, non-technical skills, and theoretical knowledge acquisition. The large effect sizes observed across domains highlight the educational relevance of structured, hands-on simulation even within a short-term intervention.

These findings support integrating simulation-based methodologies into undergraduate orthopedic education, particularly in contexts with limited clinical exposure and increasing procedural complexity. While further research is required to evaluate long-term retention and transfer to clinical performance, simulation-based learning appears to represent a valuable adjunct to traditional apprenticeship models, contributing to competency development and improved preparedness for clinical training.

## Data Availability

Data and materials can be provided at reasonable requests to the corresponding author.
