# Supplementary material for: Educational impact of a structured simulation-based orthopedic training program on technical and non-technical competency development: a prospective pre–post study
Source: J Med Life. 2026 Feb;19(2):127–35. doi: 10.25122/jml-2026-0021 (PMC13059458; doi:10.25122/jml-2026-0021)
Supplement: Supplementary file 1 [file JMedLife-19-127-s001.pdf]

Q1. The primary purpose of fracture immobilization is to:

A. Reduce pain only
B. Prevent further tissue damage and maintain fracture alignment
C. Eliminate the need for surgical treatment
D. Allow immediate mobilization

Correct answer: B

Q2. Before applying a plaster splint, which assessment is mandatory?

A. Measurement of limb circumference
B. Neurovascular assessment of the affected limb
C. Skin disinfection only
D. Radiological confirmation in all cases

Correct answer: B

Q3. Which of the following is an absolute indication for immediate fracture immobilization?

A. Closed fracture without pain
B. Suspected fracture with neurovascular compromise
C. Minor soft tissue injury
D. Chronic degenerative joint disease

Correct answer: B

Q4. The correct anatomical order of tissue layers during surgical dissection is:

A. Muscle – skin – fascia
B. Skin – subcutaneous tissue – fascia – muscle
C. Fascia – skin – muscle
D. Bone – muscle – skin

Correct answer: B

Q5. The main objective of fracture reduction is to:

A. Achieve anatomical alignment of bone fragments
B. Shorten the operative time
C. Reduce postoperative pain only
D. Avoid immobilization

Correct answer: A

Q6. The primary purpose of osteosynthesis using plates and screws is to:

A. Replace bone tissue
B. Achieve stable fixation and allow bone healing
C. Avoid surgical exposure of the fracture site
D. Eliminate the need for postoperative rehabilitation

Correct answer: B

Q7. Which of the following is a potential complication of inadequate fracture immobilization?

A. Faster bone healing
B. Increased fracture stability
C. Neurovascular injury and malunion
D. Improved patient comfort

Correct answer: C

Q8. Which suturing technique is most appropriate for the rapid closure of a linear surgical wound?

A. Simple interrupted suture
B. Continuous (running) suture
C. Mattress suture
D. Subcuticular suture

Correct answer: B

Q9. An essential principle of surgical wound closure is to:

A. Apply maximum tension to wound edges
B. Close deeper anatomical layers before the skin
C. Leave all wounds partially open
D. Avoid irrigation of the wound

Correct answer: B

Q10. When applying a plaster splint, which principle is most important to prevent complications?

A. Applying the splint as tightly as possible
B. Immobilizing only the fracture site
C. Immobilizing the joints above and below the fracture
D. Removing padding to improve stability

Correct answer: C

© 2026 by the authors. This article is an open access article distributed under the terms and conditions of the Creative Commons Attribution (CC BY 4.0) license.
